# What Does an Airline Traveler Have in Common with a Glowing Fish?

**DOI:** 10.1371/journal.pbio.0030046

**Published:** 2005-02-01

In William Gibson's novel *Pattern Recognition*, the protagonist posits a theory of jet lag: “Souls can't move that quickly, and are left behind, and must be awaited, upon arrival, like lost luggage.”

Science has yet to address the issue of a spiritual speed limit, but it is generally accepted that jet lag actually results from the upset of the body's circadian clock, a biochemical pacemaker that dictates daily rhythms in sleep and wakefulness as well as body temperature and metabolic activity. In humans, the circadian rhythm responds to many factors, but daytime–nighttime (or, more precisely, light–dark) cycles are one major regulator. It is possible to gradually reset an upset circadian clock; if travelers remain in the same place for long enough, their circadian rhythm will eventually adjust to the new time zone and ambient light patterns, and the symptoms of jet lag will disappear.

The more scientists know about the workings of the circadian clock, the closer they can come to manipulating it. Much is known about the molecular machinery of the circadian clock in the fruitfly, Drosophila melanogaster. Two circadian proteins, Clock and Cycle, cooperate to induce expression of two other proteins, Per and Tim, and when levels of Per and Tim are high enough, they cooperate to shut off their own expression. This negative feedback loop leads to periodic fluctuations in the level of Per and underlies the circadian rhythm in flies. However, until recently, not much was known about the mechanics of the circadian clock in vertebrates.[Fig pbio-0030046-g001]

**Figure pbio-0030046-g001:**
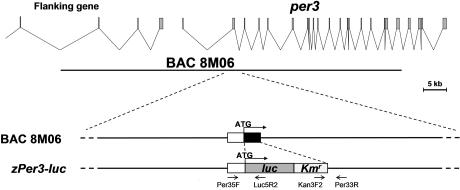
A fusion gene (period3-luciferase) was used to track circadian rhythms

Maki Kaneko and Gregory Cahill have created a new tool for investigating the components of the circadian clock in vertebrates: a zebrafish that luminesces (glows) in sync with the periodicity of its circadian clock. To do this, the researchers created a transgene that places expression of the firefly *luciferase* gene under the control of the promoter of the zebrafish circadian gene *period3 (per3)*. Each cell of the transgenic fish has one normal copy of the *per3* gene and one copy of the *period3-luciferase* fusion gene *(per3-luc)*. Therefore, whenever *per3* expression is normally turned on in a cell, the cell produces Per3 protein and also produces the luciferase protein.

While characterizing their transgenic zebrafish, the authors made some interesting findings. First, contrary to earlier studies, the authors found that *per3* periodicity is not hardwired into zebrafish embryos; instead, *per3* periodicity is entrained by alternating light–dark cycles, which must occur at specific stages in early development. Also, other external factors such as ambient temperature can influence both the level of *per3* mRNA expressed in the animal and the magnitude of its protein-level oscillations. Because the establishment of circadian rhythms in the adult animal can be so strongly influenced by conditions experienced by the embryos, the authors suggest using a standardized set of conditions for the culture of transgenic embryos in future experiments involving adult fish.

Under these controlled conditions, Kaneko and Cahill anticipate that these transgenic zebrafish will be quite useful in examining the molecular machinery of the vertebrate circadian clock. For example, researchers can use the *per3-luc* transgenic zebrafish in forward genetic screens (where researchers mutagenize the animal to induce a desired phenotype and then identify the mutated gene responsible for the phenotype). In this case, mutagenized zebrafish could be examined for disruptions of *per3-luc* periodicity or expression. What is more, luminescence can be measured quickly and noninvasively, making this animal an ideal candidate for high-throughput screening aimed at identifying components of the circadian clock in the zebrafish. Thanks to luminescent fish, scientists may someday gain enough insight to make jet lag a thing of the past.

